# Effective Dose Estimations for the Swiss Survey on Medical Radiation Exposure of the Population in 2023

**DOI:** 10.1097/HP.0000000000002135

**Published:** 2026-03-06

**Authors:** Eleni Theano Samara, Barbara Ott, Dorothea Dagassan-Berndt

**Affiliations:** 1 Radiation Protection Unit, Zurich University Hospital, University of Zurich, Zurich; 2 Radiation Protection Division, Federal Office of Public Health, Bern; 3 Dental Imaging, University Center for Dental Medicine Basel UZB, University of Basel, Basel Switzerland.

**Keywords:** effective dose, medical radiation, radiation safety, x rays

## Abstract

To estimate the annual population dose from medical examinations, the frequency of the examinations and the effective dose (E) for each examination needs to be estimated. In the last Swiss survey covering 2023, the dose per capita due to medical irradiation was estimated to be 1.7 mSv. In the framework of the 2023 survey, the E for x-rays’ modalities were calculated using different methods (bibliographic review, data from a dose management system, data from older surveys, etc.). This work aims to present the methodology followed for the estimation of E for various modalities. E was estimated for approximately 60 radiological examinations and groups of examinations. The advantages and limitations for each method are discussed.

## INTRODUCTION

Surveys for the exposure of the population to medical irradiation are organized every 5 to 10 y. Switzerland has a long tradition in this field with the first survey being published in 1961 and the last survey regarding medical exposure for examinations being performed for the year 2023 (Aroua et al. 2002; Samara et al. 2026). However, the last time that the effective dose (E) for each examination was estimated was during the Swiss survey on medical exposure in 2008 (Samara et al. 2012). In the population survey of 2013, a dose vector was calculated for each modality by multiplying the frequency of radiological sessions for one modality by the E values estimated in 2008 values and bibliographic data (Le Coultre et al. 2015). In the 2018 survey, the E for certain examinations were based on the 2013 results apart from the CT doses, which were the mean dose from different dose management systems (DMS) in the country (Bize et al. 2020). As indications, technology and techniques may have evolved since then, and an evaluation of the E was judged necessary.

The present work was carried out within the framework of a national survey on the estimation of the radiation exposure of the Swiss populations in 2023 by various radiological examinations performed in diagnostic and interventional radiology. Its aim is to present E estimation methods and summarize the E for the medical examinations and procedures included in the 2023 survey.

## MATERIALS AND METHODS

Various sources of data were used to define the E for each modality:

- Dosimetric data published for diagnostic reference levels (DRL) definition (use of median values when available);
- Dosimetric data collected by the Federal Office of Public Health (FOPH);
- Literature review, including previous national and international surveys;
- DMS data of one university hospital; and
- Dental survey carried out within the framework of the Swiss 2023 survey.

A comprehensive bibliography review was conducted with the terms "**“***radiation effective dose” radiology NOT “radiation therapy” NOT “occupational” NOT “staff” NOT “stereotactic”* for articles published between 2020 and 2024. E values from the 2008, 2013, and 2018 surveys in Switzerland were used for comparison. Additionally, the UNSCEAR report 2020/2021 (UNSCEAR 2020) with data from different Member States of the United Nations, the NCRP report 184 (NCRP 2019) on the national population survey on medical exposure of the United States, and the Austrian publication on the national survey of 2020 (Wachabauer et al. 2020) were used.

Data from a DMS of a university hospital were used. The data included dose values for procedures performed between 01 January 2023 and 30 September 2024. The E for examinations and procedures were collected directly from DMS. The DMS estimates the effective dose (E) for the radiological examinations and procedures according to the following relationship:


E=CC·Dose, (1)

where *CC* = age and body region-specific conversion coefficient and *Dose* is the dose metric for each specific modality (for example, air kerma area product for conventional radiography or interventional procedures, etc.). The CC that were used are based on ICRP 103 (ICRP 2007a) and on simulations performed with the ImpactScan dosimetry tool for the settings of a typical examination for a standard adult patient (Qaelum 2024). The E was based on the median values of the different dose metrics to better represent typical medical examinations and procedures. The final E provided in this study concerns the whole examination or procedure; i.e. for conventional radiography, the E of the examinations included the average number of projections per examination, and for CT examination it included the average number of scans per examination.

## RESULTS

### Conventional radiography (XR)

The E for conventional radiological examinations can be found in Table 1. The proposed values are on examination level. The average number of projections per examination and the conversion coefficients are also listed. Since very few data were available in the bibliography, the DMS data were used to define the different examinations as well as their corresponding effective dose. Bibliographic data were used for verification wherever available. The E for all examinations was lower than the previous surveys. The methodology followed in the 2013 and 2018 surveys differed slightly from the ones in 2008 and 2023, as examinations were grouped in more generic groups. For example, the E for skull radiography was estimated at 0.3 mSv in 2018, which corresponds to the E of all radiographies as estimated in the 2013 survey.

**Table 1.** Mean effective dose used in the Swiss survey 2023 for conventional radiography examinations. The table provides E calculated from a DMS and bibliographic review.^a^

Examination	E (mSv)	CC (mSv Gy^-1^ cm^-2^)	E (mSv) DMS estimation	E (mSv) Literature review	E (mSv) from other surveys
Skull	0.023	0.05	0.023 (incl. 2.02 projections/examination)		CH 2008: 0.04-0.1 (Aroua et al. 2011) CH 2013: 0.32 (generic for all radiography examinations) (Le Coultre et al. 2015) CH 2018: 0.3 (Bize et al. 2020) USA 2019: 0.14 (NCRP 2019) UNSCEAR 2020: 0.15 (soft tissue), 0.08 (skull/facial bones) (UNSCEAR 2020/2021 Report 2020)
Thorax	0.035	0.212	0.035 (incl. 1.89 projections/examination)	0.01-0.03 (Aksit and Olgar 2023; Bushra et al. 2023; Dolenc et al. 2022)	CH 2008: 0.05 (Aroua et al. 2011) 0.13 (CS), 0.45 (TS), 1.01 (LS) CH 2013: 0.32 (Radiography) (Le Coultre et al. 2015) CH 2018: 0.045 (Bize et al. 2020) USA 2019: 0.1 (NCRP 2019) UNSCEAR 2020:0.08 (UNSCEAR 2020) AU 2019: 0.08 (Wachabauer et al. 2020)
Abdomen	0.230	0.202	0.230 (incl. 1.31 projections/examination)	0.02-0.5 (Aksit and Olgar 2023; Alzyoud et al. 2022, 2023)	CH 2008: 0.75 (Aroua et al. 2011) CH 2013: 0.32 (Radiography) (Le Coultre et al. 2015) CH 2018: 1.15 (Bize et al. 2020) USA 2019: 0.6 (NCRP 2019) UNSCEAR 2020: 0.61(UNSCEAR 2020) AU: 0.5 (Wachabauer et al. 2020)
Pelvis/Pelvis (soft tissue)/Hip	0.330	0.202	0.330 (incl. 1.92 projections/examination)	0.19-0.85 (Dolenc et al. 2022)	CH 2008: 0.45-0.9 (Aroua et al. 2011) CH 2013: 0.32 (Radiography) (Le Coultre et al. 2015) CH 2018: 1.15 (Bize et al. 2020) USA 2019: 0.4 (NCRP 2019) UNSCEAR 2020: 0.49 (UNSCEAR 2020) AU 2019: 0.5 (Wachabauer et al. 2020)
Extremities	0.001	0.01 (upper extremities) 0.005 (lower extremities)	0.001 (This is the weighted effective dose of various examinations of the extremities: hand, foot, knee, upper ankle joint (OSG), wrist, elbow, lower leg, upper leg, forearm, upper arm. For this reason, no information can be provided on the number of images per examination.)	0.00045-0.03 (Dolenc et al. 2022; Singh et al. 2022)	CH 2008: 0.0004-0.02 (Aroua et al. 2011) CH 2013: 0.32 (Radiography) (Le Coultre et al. 2015) CH 2018: 0.01 (Bize et al. 2020) USA 2019: 0-0.006 (NCRP 2019) UNSCEAR 2020: 0.02 (UNSCEAR 2020)
Spine	0.402 (weighted average for CS, TS, LS)	0.212 (CS) 0.212 (TS) 0.202 (LS)	0.05 (CS), 0.19 (TS), 0.66 (LS), (incl. 2.15 (CS), 2.0 (TS), 2.05 (LS) projections/examination)	0.1-0.4 (Bradley & Snaith 2023; Bushra et al. 2023; Dolenc et al. 2022)	CH 2008: 0.1 (CS), 0.5 (TS), 1.5 (LS) (Aroua et al. 2011) CH 2013: 0.32 (Radiography) (Le Coultre et al. 2015) CH 2018: 1.6 (Bize et al. 2020) USA 2019: 0.2 (CS), 1.0 (TS), 1.4 (LS) (NCRP 2019) UNSCEAR 2020: 0.13 (CS), 0.45 (TS), 1.01 (LS) (UNSCEAR 2020) AU 2019: 0.5 (Wachabauer et al. 2020)

a Abbreviations: CH: Switzerland, USA: United States of America, AU: Austria, UNSCEAR: United Nations Scientific Committee on the Effects of Atomic Radiation, CS: cervical spine, TS: thoracic spine, LS: lumbar spine.

### Fluoroscopy examinations

The E for fluoroscopy examinations can be found in Table 2. Very few data were available in the bibliography, thus the DMS data were used primarily to define the different examinations and the corresponding E. Bibliographic data were used to complete the list of missing examinations and for data validation. One particular case was the general category, “Conventional fluoroscopy (various),” which included fluoroscopy examinations that could not be attributed in more specific category. For the needs of this study, an average of 2.8 mSv was determined for “Conventional fluoroscopy (various).” Differences were observed in the previous surveys performed in the country. In the 2008 survey, separate dose values were determined for the individual conventional fluoroscopy procedures with a mean value for these individual procedures of 3.5 mSv, and a weighted mean value of 2.7 mSv. In the 2013 and 2018 surveys, an effective dose of 8 mSv was used for one general category of fluoroscopy procedures.

**Table 2.** Mean effective dose used in the Swiss survey 2023 for fluoroscopy examinations. The table provides E calculated from a DMS and bibliographic review.

Fluoroscopy examination	E (mSv)	CC (mSv/Gy cm^-2^)	E (mSv) DMS estimation	E (mSv) Literature review	E (mSv) from other surveys
ERCP	3.9	0.202	3.9		CH 2008: 5.49 (Aroua et al. 2011) UNSCEAR 2020: 4.9 (UNSCEAR 2020) USA 2019: 2 (biliary incl. ERCP) (NCRP 2019)
Cystography	0.2	0.202	0.2		CH 2008: 1.8 (Aroua et al. 2011)
Defecography	8.8				CH 2008: 12 (Aroua et al. 2011) UNSCEAR 2020: 8.8 (UNSCEAR 2020)
Ba swallow	0.32			0.32 (Tipnis et al. 2022)	CH 2008: 1.1 (Aroua et al. 2011)
Myelography	4.75				CH 2008: 0.6-11 (Aroua et al. 2011) UNSCEAR 2020: 5.5 (UNSCEAR 2020) USA 2019: 4 (NCRP 2019)
Cholangiography (other)	0.8	0.202	0.8		CH 2008: 1.8-8.1 (Aroua et al. 2011) UNSCEAR 2020: 8.5 (UNSCEAR 2020)
Urogenital (various)	1.6	0.202	0.3-2.7 (with 1.2 mean value of various urogenital examinations)		CH 2008: 1.1-3.5 (Aroua et al. 2011) UNSCEAR 2020: 1.6 (UNSCEAR 2020) USA 2019: 2 (NCRP 2019)
Gynecological (various)	1				CH 2008: 2.5 (Aroua et al. 2011) USA 2019: 1 (NCRP 2019)
Arthrography	2.1				CH 2008: 0.003-0.8 (Aroua et al. 2011) UNSCEAR 2020: 2.1 (UNSCEAR 2020) USA 2019: 0.2 (NCRP 2019)
Gastrointestinal (GI) Colon	8			Ba follow: 7.5 (Golikov et al. 2024)	CH 2008: 12 (Aroua et al. 2011) USA 2019: 6 (NCRP 2019) AU 2019 (Ba meal, Ba enema, Ba follow, IVU): 4 (Wachabauer et al. 2020)
Conventional fluoroscopy (various)	2.8				CH 2013: 8 (Le Coultre et al. 2015) CH 2018: 8 (Bize et al. 2020) AU 2019: 5 (Wachabauer et al. 2020) USA 2019: 4 (NCRP 2019)
GI various	3.4				CH 2008: 3.8-7 (Aroua et al. 2011) UNSCEAR 2020: 3.4 (UNSCEAR 2020) AU 2019 (Ba meal, Ba enema, Ba follow, IVU): 4 (Wachabauer et al. 2020)
Intravenous Urography (IVU)	2.4			1.51 (Hsieh et al. 2022)	UNSCEAR 2020: 2.4 (UNSCEAR 2020) AU 2019 (Ba meal, Ba enema, Ba follow, IVU): 4 (Wachabauer et al. 2020)
Retrograde pyelography	0.9	0.202	0.9		CH 2008: 2.3 (Aroua et al. 2011)
Retrograde urethrography	0.4	0.202	0.4		CH 2008: 1.1 (Aroua et al. 2011) USA 2019: 3 (NCRP 2019)

### Diagnostic and therapeutic interventional procedures

The E for interventional procedures (diagnostic and therapeutic) was determined by using mainly DMS data. The only exception was coronary angiography (CA), where the dose value used was the value used in three surveys (NCRP 2019; UNSCEAR 2020; Wachabauer et al. 2020). The reason for this was that CA (diagnostic examination) was always recorded together with Percutaneous Coronary Intervention (PCI; therapeutic procedure) in the DMS used; that would lead to an overestimation of the E due to the therapeutic part. The remaining examinations in the DMS could be clearly distinguished from each other. The effective doses for “angiography, abdominal/pulmonary/pelvic/cerebral/other” and “angioplasty (except PCI)” were calculated as the mean value of various procedures. The values are shown in Table 3.

**Table 3.** Mean effective dose used in the Swiss survey 2023 for diagnostic and therapeutic interventional procedures. The table provides E calculated from a DMS and bibliographic review.

Procedure	E (mSv)	E (mSv) DMS estimation	CC (mSv/Gy cm^-2^)	E (mSv) Literature review	E (mSv) from other surveys
Interventional procedure - Diagnostic procedures					
Coronarography (CA)	7.00			4.75-20.4 (Uniyal et al. 2024) (Golikov et al. 2024)	CH 2008: 11.2 (Aroua et al. 2011) CH 2013: 14 (Le Coultre et al. 2015) CH 2018: 14 (Bize et al. 2020) AU 2019: 7.0 (Wachabauer et al. 2020) UNSCEAR 2020: 7.0 (UNSCEAR 2020) USA 2019: 7 (NCRP 2019)
Angiography lower extremities	3.10	3.1	0.02		CH 2008: 8 (Aroua et al. 2011) UNSCEAR 2020: 3.2 (UNSCEAR 2020) USA 2019: 1 (NCRP 2019)
Angiography upper extremities	0.11	0.11	0.01		CH 2008: 8 (Aroua et al. 2011) UNSCEAR 2020: 3.2 (UNSCEAR 2020)
Angiography abdomen, thorax, pelvis, cerebral, other	9.20	4-31 (with a mean value of 9.2 for various angiographies)	0.05 (Cerebral) 0.212 (Thorax) 0.202 (Abdomen/Pelvis)		CH 2008: 1-8 (Aroua et al. 2011) CH 2013: 8 (Le Coultre et al. 2015) CH 2018: 8 (Bize et al. 2020) UNSCEAR 2020: 6.9 (cerebral angiography) 4.8 (thoracic angiography), 8.0 (abdominal angiography), 7.5 (pelvic angiography) (UNSCEAR 2020) USA 2019: 1-27 (NCRP 2019)
Right heart angiography	1.10	1.1	0.24		
Interventional procedure - Therapeutic procedures					
Percutaneous Coronary Interventions (PCI)	20.6	20.6	0.24		CH 2008: 17 (Aroua et al. 2011) CH 2013: 20 (Le Coultre et al. 2015) CH 2018: 20 (Bize et al. 2020) AU 2019: 17.0 (Wachabauer et al. 2020) USA 2019: 23-30 (NCRP 2019)
Angioplasty (other than PCI)	5	0.7-83.2 (with a mean value of 5.0 for various angioplasties)	0.05 (Cerebral) 0.212 (Thorax) 0.202 (Abdomen/Pelvis)		CH 2013: 20 (Le Coultre et al. 2015) USA 2019: 0.2-55 (NCRP 2019)
Pacemaker/Defibrillator	2.65	2.6	0.24		CH 2008: 2.8 (Aroua et al. 2011) USA 2019: 1 (NCRP 2019)
Electrophysiology (EP)	2.6	2.6	0.24		CH 2008: 3.3 (Aroua et al. 2011) USA 2019: 3.2 (NCRP 2019)
Heart valves	1.9	1.9	0.24		CH 2008: 2.93 (Aroua et al. 2011)
Pain management procedures	0.5	0.5	0.21		USA 2019: 1 (Spine injections) (NCRP 2019)

The values for interventional procedures can vary considerably depending on the country, clinic, and patient. This may be due to differences in patient anatomy, procedure complexity, irradiated body region, puncture site (femoral/radial), x-ray device and protocols used, operator experience, accuracy in procedure definition (angiography with or without subsequent angioplasty), the Monte Carlo simulation for E-calculation, etc. For example, the effective dose for angiography of the upper extremities was determined at 0.11 mSv based on data from a specific angiology device connected to the DMS, while literature indicated a significantly higher value (UNSCEAR 2020). The same applies to angioplasties other than PCI. For these procedures, the range of effective doses was particularly wide, ranging from 0.7 to 83.2 mSv, with a mean value for this category of 5 mSv. Lower E values are expected for extremities and higher E values for angioplasty on the trunk, such as endovascular procedures for abdominal aortic aneurysms.

### Computed tomography

The FOPH provided dosimetric data on the expected evolution of the E values based on a survey run in 2023 in various hospitals (Ott et al. 2025). The percent change was applied on the E values from the survey 2018 (Table 4). A verification was run with the DMS data of the university hospital. To take into account that different protocols lead to different E after examination, protocols were grouped under the same examination. For example, the head examinations included protocols with head native, head native+head with contrast medium, head native+head angiography, head native+perfusion+head angiography, etc. Similarly, the chest examination includes thorax native, thorax inspiration+thorax expiration, calcium score, etc. The E for CT intervention is the average dose from literature review. The E dose for “CT other” and “CT angiography” is the mean value of the E of all examinations, as both categories may refer to all body regions. The range of E provided by recent publications can also be found in Table 4.

**Table 4.** Mean effective dose used in the Swiss survey 2023 for CT examinations. The table provides E calculated from a DMS and bibliographic review.^a^.

CT Examination	E (mSv)	E (mSv) DMS estimation	E (mSv) Literature review	E (mSv) from other surveys
Brain	2.40	2.4	0.68-6.25 (Akyea-Larbi et al. 2024; Altmann et al. 2023; Bazoma et al. 2022; Golikov et al. 2024; Jeyasugiththan et al. 2023; Khoramian et al. 2024; Lenfant et al. 2022; Lu et al. 2022; Ucar et al. 2024)	CH 2008: 2.6 (Aroua et al. 2011) CH 2018: 2.36 (Le Coultre et al. 2015) USA 2019: 1.6 (Bize et al. 2020) UNSCEAR 2020: 1.9 (UNSCEAR 2020) AU 2019: 1.5 (Wachabauer et al. 2020)
Skull/Facial bones	2.10			CH 2008: 0.5-1.55 (Aroua et al. 2011) CH 2018: 2.36 (Bize et al. 2020) UNSCEAR 2020: 1.5 (UNSCEAR 2020) AU 2019: 1.5 (Wachabauer et al. 2020)
Neck	2.10			CH 2008: 2.57 (Aroua et al. 2011) CH 2018: 2.1 (Bize et al. 2020) USA 2019: 1.2 (NCRP 2019) UNSCEAR 2020: 2.8 (UNSCEAR 2020) AU 2019: 1.5 (Wachabauer et al. 2020)
Thorax	3.50	2.9	0.78-8.1 (Akyea-Larbi et al. 2024; Chen et al. 2024; Daniel et al. 2023; Demircioglu et al. 2024; Golikov et al. 2024; Hagar et al. 2023; Jamshidi et al. 2023; Shim et al. 2023; Tonkopi et al. 2024; Yang et al. 2022)	CH 2008: 5.76 (Aroua et al. 2011) CH 2018: 3.8 (Bize et al. 2020) USA 2019: 6.1 (NCRP 2019) UNSCEAR 2020: 6.4 (UNSCEAR 2020) AU 2019: 5.7 (Wachabauer et al. 2020)
Upper abdomen/Abdomen	9.90	7.8 (Tho-Abd) 5.8 (Abd)	6.7-28.7 (Akyea-Larbi et al. 2024; Bazoma et al. 2022; Dalah et al. 2024; Daniel et al. 2023; Golikov et al. 2024; Hsieh et al. 2022; Jamshidi et al. 2023; Jansen et al. 2023)	CH 2008: 9.26 (Aroua et al. 2011) CH 2018: 10.5 (Bize et al. 2020) USA 2019: 7.7 (NCRP 2019) UNSCEAR 2020: 11.2 (abdomen), 10.3 (liver, pancreas, kidneys) (UNSCEAR 2020) AU 2019: 13.0 (Wachabauer et al. 2020)
Pelvis/SIJ	7.00		1.16-21.6 (Euler et al. 2023; Gassenmaier et al. 2022; Grunz et al. 2023; Kunz et al. 2023; Liang et al. 2023)	CH 2008: 5.7-14.3 (Aroua et al. 2011) CH 2018: 7.9 (Bize et al. 2020) USA 2019: 7.7 (NCRP 2019) UNSCEAR 2020: 8.8 (pelvic bone), 10.9 (pelvic soft tissue/vascular), 5.0 (pelvimetry) (UNSCEAR 2020) AU 2019: 13.0 (Wachabauer et al. 2020)
Spine	11.40			CH 2008: 16.7 (Aroua et al. 2011) CH 2018: 10.7 (Bize et al. 2020) USA 2019: 8.8 (NCRP 2019) UNSCEAR 2020: 3.1 (CS), 8.0 (TS), 9.4 (LS), 14.5 (FS) (UNSCEAR 2020) AU 2019: 6.0 (Wachabauer et al. 2020)
Shoulder/Upper arm	5.80			CH 2008: 1.8 (Aroua et al. 2011) CH 2018: 5.8 (Bize et al. 2020) USA 2019: 1.7 (upper extremities) (NCRP 2019) UNSCEAR 2020: 2.1 (UNSCEAR 2020)
Elbow/Lower arm	3.20			CH 2008: 1.8 (Aroua et al. 2011) CH 2018: 3.2 (Bize et al. 2020) USA 2019: 1.7 (upper extremities) (NCRP 2019) UNSCEAR 2020: 2.1(UNSCEAR 2020)
Wrist/Hand	1.90			CH 2008: 1.8 (Aroua et al. 2011) CH 2018: 1.9 (Bize et al. 2020) USA 2019: 1.7 (upper extremities) (NCRP 2019) UNSCEAR 2020: 3.2 (UNSCEAR 2020)
Hip/Upper leg	9.30			CH 2008: 5.7 (Aroua et al. 2011) CH 2018: 11 (Bize et al. 2020) USA 2019: 3.2 (lower extremities) (NCRP 2019) UNSCEAR 2020: 2.1 (UNSCEAR 2020)
Knee/Lower leg	2.70			CH 2008: 0.1 (Aroua et al. 2011) CH 2018: 2.7 (Bize et al. 2020) USA 2019: 3.2 (lower extremities) (NCRP 2019) UNSCEAR 2020: 2.1 (UNSCEAR 2020)
Foot/Ankle	0.06			CH 2008: 0.1 (Aroua et al. 2011) CH 2018: 0.06 (Bize et al. 2020) USA 2019: 3.2 (lower extremities) (NCRP 2019) UNSCEAR 2020: 2.1 (UNSCEAR 2020)
Intervention	8		0.5-16.8 (Alexander et al. 2024; Bronnimann et al. 2024; Burgard et al. 2021; Cahalane et al. 2023; Kamp et al. 2023; Ren et al. 2022; Stahl et al. 2024; Trumm et al. 2022; Zlevor et al. 2023)	CH 2008: 3.9 (Aroua et al. 2011) USA 2019: 5.0 (lower extremities) (NCRP 2019)
Angiography	5			CH 2018: 3.9 (Bize et al. 2020) USA 2019: 5.1 (NCRP 2019)
Other	5			CH 2013: 8.54 (CT Scanning) (Le Coultre et al. 2015)

a Abbreviations: CH: Switzerland, USA: United States of America, AU: Austria, UNSCEAR: United Nations Scientific Committee on the Effects of Atomic Radiation, CS: cervical spine, TS: thoracic spine, LS: lumbar spine, FS: full spine.

Regarding the contribution of the CT part to PET/CT and SPECT/CT, the E/dose length product (DLP) coefficients (mSv mGy^-1^ cm^-1^) were adopted from the ICRP publication 102 (ICRP 2007b). The DLP values were adopted from the national DRL survey on hybrid imaging in nuclear medicine (Lima et al. 2018). The dose length products used here represent weighted median values according to the objective of the examination (diagnostics, D, or attenuation correction/localization, AC-L). For PET/CT tumor and SPECT/CT skeletal examinations, additional weighting is applied using the relevant protocols (see Table 5, column “Comments”). The weights are based on the examination frequencies specified in Lima et al. (2018).

**Table 5.** Mean effective dose used in the Swiss survey 2023 for CT part of nuclear medicine examinations. The table provides the conversion coefficients used for the calculation of E for CT examinations as well as the corresponding DLP values.^a^

Examination	CC (mSv/mGy cm^-1^)	DLP (mGy cm^-1^)	E (mSv)	Comments
PET/CT Oncology	0.015	394.8	**5.92**	PET Oncology whole-body, torso, abdomen, upper abdomen (CT AC-L)
PET/CT Myocardial	0.014	40	**0.56**	PET-CT Myocardial (D and AC-L)
PET/CT Head	0.0031	79	**0.25**	PET-CT Dementia Head (D and AC-L)
SPECT/CT Bones	0.015	217.4	**3.26**	SPECT Bone-Pelvis, Bone-Spine, Bone extremities (D and AC-L)
SPECT/CT Thyroid	0.0059	177	**1.04**	SPECT Thyroid-Parathyroid (D and AC-L)
SPECT/CT Ventilation/Per-fusion	0.014	92	**1.28**	SPECT Lung Ventilation/Perfusion (D und AC-L)

a Abbreviations: AC-L: Attenuation correction and localization CT, D: Diagnostic CT.

### Dental radiology

The effective dose for the cone beam CT (CBCT) examinations depends on the field of view, voxel-size and exposure parameter. Data regarding the field-of-view (FOV) was collected through the dental survey conducted in the framework of the Swiss survey 2023. For the calculation needs, it was assumed that the small FOV was 5 × 5 cm^2^, the medium 8 × 8 cm^2^ and the large 15 × 15 cm^2^. The normalized dose area product (DAP) for all examination was 421 mGy cm^-2^ for a FOV of 5 × 5 cm^2^, as it was estimated in the survey in 2019 (Deleu et al. 2020). We assumed an application of 90 kVp for all cases. The conversion coefficients were adopted from the review of Mah et al. (2021). The weighted E for CBCT examinations was estimated at 0.13 mSv, taking into account the frequency of the FOV resulted from the 2023 dental survey (Table 6). The values are in coherence with literature, where the values ranged between 0.005 to 1.1 mSv (Colceriu-Simon et al. 2019; Kaasalainen et al. 2021; Lee and Badal 2021; Qiang et al. 2019; Santos et al. 2024; Soderqvist et al. 2023), while the dose for CBCT for the Swiss survey 2018 was 0.2 mSv.

**Table 6.** Mean effective dose used in the Swiss survey 2023 CBCT examinations. The table provides the conversion coefficients used for the calculation of E for the CBCT examinations as well as the corresponding parameters. The % frequency is the frequency of the CBCT examinations with small/medium/large FOV according to the dental survey 2023.

	FOV (length×height in cm)	CC (μSv/mGy cm^-2^ kV)	DAP (mGy cm^-2^)	kV	FOV (cm^2^)	E (mSv)	% frequency
Small FOV	4 × 4, 5 × 5, 6 × 6, 8x5	0.0015	421	90	5x5	**0.06**	**0.49**
Medium FOV	5 × 10, 8 × 8, 8 × 9	0.0014	421	90	8x8	**0.14**	**0.29**
Large FOV	10 × 10, 15 × 15, 17 × 23	0.0009	421	90	15x15	**0.31**	**0.22**

For the intraoral examinations, a literature review was conducted. The E in 2018 was estimated at 0.02 mSv, but most of the recent publications estimated the E for intraoral examinations lower (Dorsey et al. 2024; Johnson and Ludlow 2020; Lee and Badal 2021; Qiang et al. 2019; Shetty et al. 2019). NCRP 2019 reported a mean E of 0.001 mSv (NCRP 2019), while UNSCEAR estimated it 0.01 mSv (UNSCEAR 2020). For the Swiss survey 2023, we proposed the use of E of 0.01 mSv (Table 6).

Regarding the panoramic radiograph or orthopantomogram (OPG) examinations (Table 6), the literature review showed no major changes, so the E for 2023 could be kept at 0.02 mSv (Ahmed 2023; Dorsey et al. 2024; Lee and Badal 2021; Qiang et al. 2019; UNSCEAR 2020).

Finally, a well-established dental examination for orthodontic treatment, cephalometry was added in the survey of 2023. The effective dose was conservatively set at 0.02 mSv based on literature (Table 6), which indicated a range of 0.002–0.02 mSv (Kissel et al. 2021; Ting et al. 2020), while the previous survey including cephalometry in CH was that of 2008 with 0.06 mSv (Aroua et al. 2011).

### Mammography

The E from screening mammography is 0.55 mSv, with the following assumptions: two views, bilateral craniocaudal (CC) at 1.10 mGy and mediolateral oblique (MLO) at 1.18 mGy of each breast (mean compressed breast tissue at 56 mm), a mean glandular dose for the entire breast tissue of 1.8 mGy per view, and a tissue-specific factor *wT* for the breast of 0.12 (ICRP 2007a) (Dupont et al. 2024), assuming that only the breast is exposed to radiation during a mammography examination. The E from diagnostic mammography is 0.66 mSv, as the diagnostic procedure also includes two bilateral views for CC and MLO, and 20% of the patients undergo one or more additional projections (Samara et al. 2019). The effective dose for mammography is higher than in previous surveys as the number of projections was “1 or 2 per breast” (Aroua et al. 2011). The technique for the screening has been standardized since 2014 (Bruetsch et al. 2014). A comparison across different surveys can also be found in Table 7.

**Table 7.** Mean effective dose used in the Swiss survey 2023 for mammography examinations. The table provides data from a DMS and E from other national surveys.

Examination	E (mSv)	E (mSv) from other surveys
Screening mammography	0.55	CH 2008: 0.16 (Aroua et al. 2011) CH 2013: 0.36 (Le Coultre et al. 2015) CH 2018: 0.36 (Bize et al. 2020) AU 2019: 0.33 (Wachabauer et al. 2020) UNSCEAR 2020: 0.52 (UNSCEAR 2020) USA 2019: 0.36 (NCRP 2019)
Diagnostic mammography	0.66	CH 2008: 0.16 (Aroua et al. 2011) CH 2013: 0.36 (Le Coultre et al. 2015) CH 2018: 0.36 (Bize et al. 2020) AU 2019: 0.33 (Wachabauer et al. 2020) UNSCEAR 2020: 0.66 (UNSCEAR 2020) USA 2019: 0.36 (NCRP 2019)

### Bone densitometry

The E for bone densitometry was set at 0.001 mSv for both lumbar spine and hip regions following literature review (NCRP 2019) (Table 8). The survey of 2008 estimated the dose for lumbar spine at 0.004 mSv and that of the hip at 0.002 mSv (2008), while they were not taken into consideration in the surveys of 2013 and 2018. In the US survey in 2019, the dose was estimated at 0.001 mSv, but the authors concluded that the dose was too low to include bone densitometry examinations with dual x-ray absorptiometry in further estimations (NCRP 2019).

**Table 8.** Mean effective dose used in the Swiss survey 2023 for bone densitometry examinations. The table provides data from other national surveys.

Examination	E (mSv)	E (mSv) from other surveys
Bone densitometry (lumbar spine, hips)	0.001	CH 2008: 0.004 (lumbar spine) (Aroua et al. 2011) CH 2008: 0.002 (hips) (Aroua et al. 2011) USA 2019: 0.001 (NCRP 2019)

## DISCUSSION

In this study, we provided the E that was used in the Swiss survey regarding the population exposure to medical radiological examinations performed in 2023. E estimations are complex due to several reasons. By definition, E includes the calculation of organ doses based on absorbed dose and the use of various computational phantoms and changing values of tissue weighting factors. Dose exposure depends on the x-ray device, examination protocol and x-ray parameters, number of projection or scans, experience of the operating physician, and complexity of the anatomy; thus, E may substantially differ from patient to patient and from center to center. When adequately covered, primary data collection by means of surveys is the best method for obtaining values that are representative of the country. However, this requires that examination protocols are clearly defined, stored in a comparable manner in the systems of all participating hospitals/institutes, and understood in the same way by all participants. In the survey 2023, different methods were used to determine E of examinations and procedures.

The bibliographic data on E are scarce, especially for radiography and fluoroscopy guided procedures. This could be attributed to reduced interest for more “traditional” examinations like radiography and the difficulty to estimate the doses for fluoroscopy guided procedures outside the radiology department. Literature includes various articles giving indications of the absorbed dose, such as DAP, DLP, etc. Their advantage is that the data are potentially extensive including rare or sophisticated examinations and procedures. National and international studies provide also various indications and E that may cover the list of examinations in a population survey. Their disadvantage, however, is that the transferability of the results to another country or even healthcare facility cannot be guaranteed.

Extraction of values from DMS would generally provide the most accurate values and the most realistic values for Switzerland. The advantage lies in the high resolution of the data (provided that the examination protocols can be distinguished from one another, data can be extracted at the examination level) and in the high degree of accuracy, both regarding Swiss conditions and the representation of current technological developments. The disadvantage was the effort involved in obtaining such data and the fact that data came from only one university hospital.

The advantage of using older surveys on diagnostic reference values (DRL) was that diagnostic reference values for different modalities are based on a broad data set and reflect the country context. On the downside, though, technology is moving fast, and the available DRL might already be out of date (especially for CT, radiographies, and interventional procedures), which in its turn will affect the dose estimations for the population survey. In addition, E may be overestimated when using the DRL as they represent the 75^th^ percentile of the distribution. The median values are appropriate for determining the dose for typical procedures. For the needs of our calculations, the median values were available from the DRL surveys and used for the calculation of E for CT (Ott et al. 2025) (including the CT part of nuclear medicine; Lima et al. 2018), for mammography (Dupont et al. 2024), and for dental CBCT examinations (Deleu et al. 2020).

## CONCLUSION

The E for approximately 60 radiological examinations and groups of examinations was estimated in the framework of the Swiss population survey. Our methodology provided a practical tool for E determination. Each of the methods listed had strengths and weaknesses. Comparing values from different sources proved useful. In this way, we ensured that the dose values determined reflect the state of practical application in Switzerland in 2023 as accurately as possible while also ensuring a certain degree of continuity over time.
